# Thalamic neuronal and bioenergetic function in painful diabetic peripheral neuropathy: a dual magnetic resonance spectroscopy study

**DOI:** 10.1007/s00125-026-06846-8

**Published:** 2026-09-10

**Authors:** Gordon Sloan, Adriana Anton, Kevin Teh, Sharon Caunt, Ancy Thomas, Iain Wilkinson, Dinesh Selvarajah, Solomon Tesfaye

**Affiliations:** 1 Diabetes Research Unit, Royal Hallamshire Hospital, Sheffield Teaching Hospitals NHS Foundation Trust, Sheffield, UK; 2 Division of Clinical Medicine, University of Sheffield, Sheffield, UK

**Keywords:** Diabetes, Diabetic neuropathy, Diabetic peripheral neuropathy, Neuropathic pain, Painful diabetic peripheral neuropathy

## Abstract

**Aims/hypothesis:**

The aim of this cross-sectional study was to investigate three imaging parameters in people with type 2 diabetes to examine the volume, neuronal function and bioenergetic status of the thalamus in painful diabetic peripheral neuropathy (DPN) compared with painless DPN.

**Methods:**

Forty-nine participants were recruited: 38 with type 2 diabetes (of whom 11 had painless DPN, nine had diabetes without DPN and 18 had painful DPN) and 11 healthy volunteers. All underwent detailed neurological phenotyping, structural brain MRI, and dual ^1^H-MRS and ^31^P-MRS during the same study visit.

**Results:**

Thalamic volume did not differ between groups. However, significant group differences were observed in neuronal function, as assessed by the *N*-acetylaspartate:choline (NAA:Cho) ratio, and in bioenergetics, as assessed by the inorganic phosphate:ATP (Pi:ATP) ratio. NAA:Cho was significantly higher in individuals with painful DPN than in those with painless DPN. The Pi:ATP ratio was significantly lower in individuals with painful DPN than in those with painless DPN. Moreover, within the painful DPN group, pain intensity correlated with thalamic Pi:ATP, and NAA:Cho significantly correlated with Pi:ATP across the whole cohort.

**Conclusions/interpretation:**

These findings suggest that an impaired thalamic neuronal and altered bioenergetic state, potentially consistent with impaired oxidative phosphorylation, exists in painless DPN, and that preservation of thalamic bioenergetic and neuronal profiles could be an important central mechanism of painful DPN. These findings may have clinical relevance as metabolites measured by ^31^P-MRS may have the potential to act as objective biomarkers of painful DPN or neuropathic pain in future clinical trials.

**Graphical Abstract:**

**Figure:**
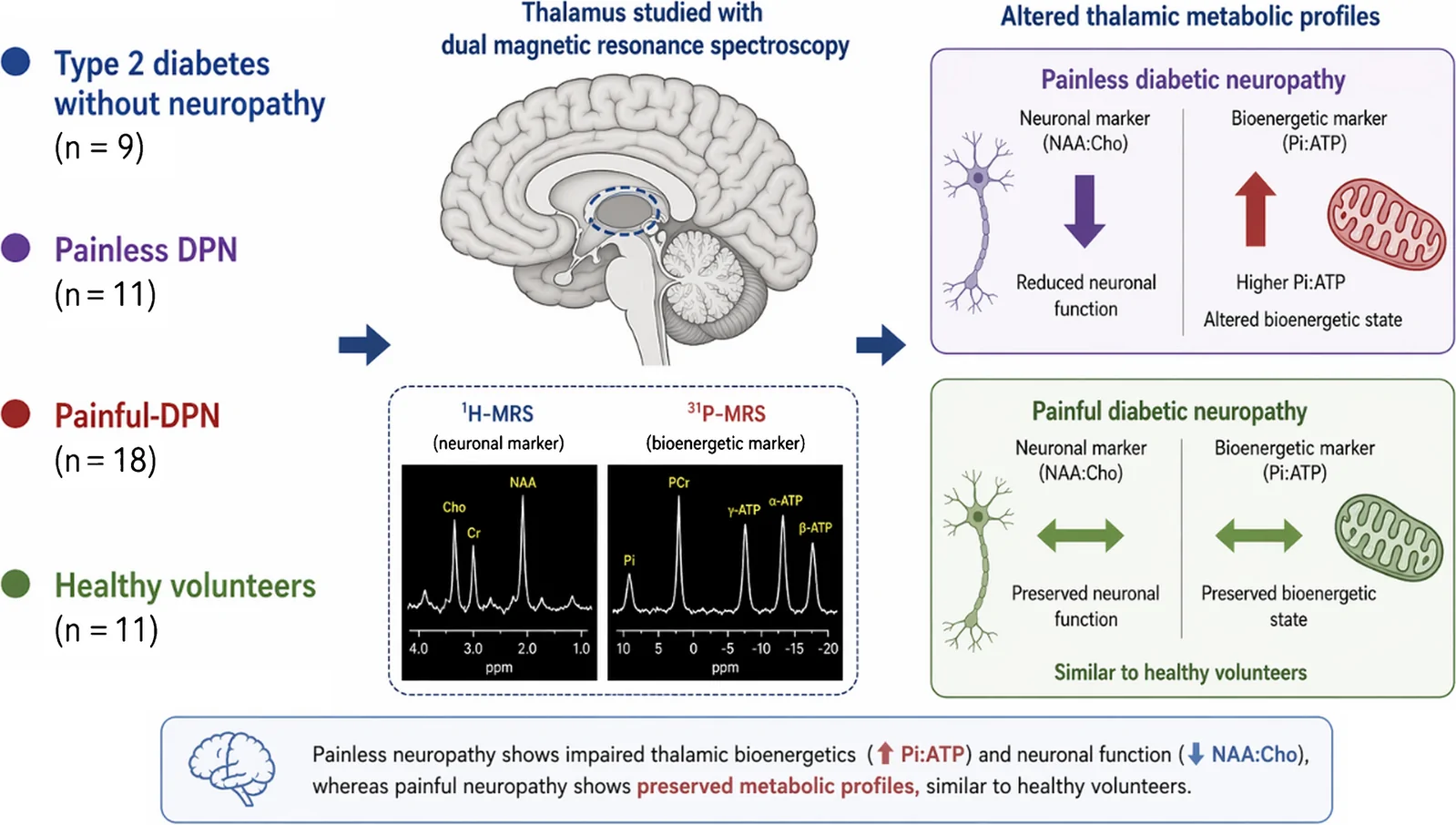

**Supplementary Information:**

The online version contains peer-reviewed but unedited supplementary material available at https://doi.org/10.1007/s00125-026-06846-8.

**Figure:**
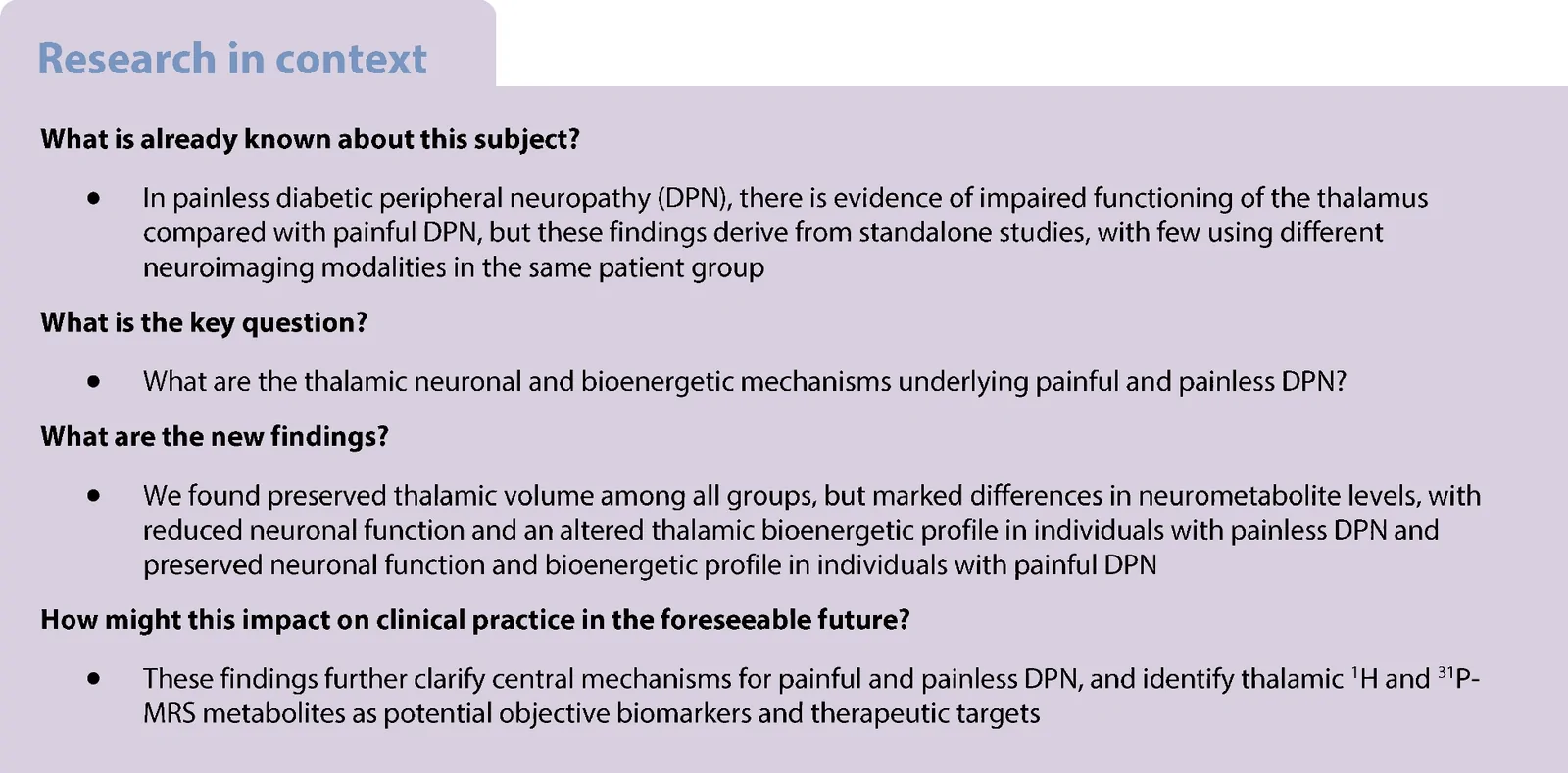

## Introduction

The global prevalence of diabetes is worryingly high, with a projected rise from 529 million in 2021 to more than 1.3 billion by 2050 [1]. Diabetic peripheral neuropathy (DPN) is a common complication, affecting up to 50% of individuals with diabetes [2], and accounts for the fifth-highest number of disability-adjusted life years (DALYs) related to nervous system disorders globally in 2021 [3]. It is associated with increased mortality, and is the major risk factor for diabetic foot ulceration, which can result in lower-limb amputation [4]. Individuals with DPN may be asymptomatic, may develop non-painful symptoms of numbness or paraesthesia (classified as having painless DPN), or may have painful neuropathic symptoms (painful DPN). Painful DPN can be distressing, often resulting in sleep impairment, mood disorders and a reduced quality of life [5]. Currently recommended treatments provide sub-optimal pain relief, with only 50% pain reduction in approximately 40% of individuals [6]. In large part, this is because the mechanisms underlying painful DPN are poorly understood, and treatments for the condition target symptoms rather than disease mechanisms [2]. There is therefore a critical need for a greater understanding of the pathophysiological mechanisms of painful DPN.

Over the past 25 years, research has demonstrated central nervous system involvement in DPN [7]. Although the neuropathic process starts in the peripheral nervous system, studies have found structural and functional changes in the spinal cord [8–10] and the brain [11–16] in both painful and painless DPN. The thalamus is a key region of the brain involved in somatosensory and pain processing, and numerous studies have shown alterations in the thalamus in individuals with DPN. The physiological function of the thalamus is to receive and modulate somatosensory input from the peripheral nervous system, then relay impulses to the somatosensory cortices and other pain-processing areas of the brain (e.g. the insular cortex, anterior cingulate cortex and dorsolateral pre-frontal cortex). In painless DPN, there is evidence of impaired functioning of the thalamus, including sluggish blood flow [17], reduced levels of the neurotransmitter γ-aminobutyric acid (GABA) [11], and reduced levels of *N*-acetylaspartate (NAA), suggesting neuronal dysfunction [14]. However, in painful DPN, there is hyperperfusion and preserved levels of GABA and NAA [11, 14, 17]. These findings derive largely from standalone studies, and few use different neuroimaging modalities in the same patient group to investigate how imaging parameters are related. In this study, for the first time, we have applied multimodal neuroimaging to assess the structure, neuronal and bioenergetic function of the thalamus in well-characterised cohorts of people with type 2 diabetes and painful or painless DPN, individuals with diabetes without DPN (no DPN) and healthy volunteers (HV). We hypothesised that thalamic neuronal and bioenergetic profiles would differ between painful and painless DPN, assessed using metabolite ratios derived from magnetic resonance spectroscopy (^1^H-MRS and ^31^P-MRS, respectively).

## Methods

### Study approval and consent

Participants gave written, informed consent. The study was approved by the Nottingham Research Ethics Committee (reference no. 17/EM/0430).

### Participants

Forty-nine right-handed participants were recruited to this cross-sectional cohort neuroimaging study: 38 with type 2 diabetes (of whom 11 had painless DPN, nine had diabetes without DPN and 18 had painful DPN) and 11 healthy volunteers. Participants with diabetes had type 2 diabetes according to the World Health Organization criteria [18], and were recruited to the study from Sheffield Teaching Hospital outpatient clinics between 2018 and 2020. The study population is representative of adults with type 2 diabetes attending a UK tertiary diabetes service. Exclusion criteria were current or past history of excessive alcohol usage (>14 units/week), non-diabetic neuropathies, other neurological disorders that may confound radiological/clinical assessments, or other factors that would preclude MRI (e.g. pacemaker/claustrophobia), pregnancy, major lower-limb amputation, use of antidepressant medication (other than for neuropathic pain treatment), recurrent hypoglycaemia and moderate-to-severe non-DPN pain. Both male and female participants were eligible, and recruitment was not restricted on the basis of sex or gender. Sex was recorded from National Health Service medical records.

### Peripheral neurological and clinical assessments

Participants underwent clinical and neurophysiological assessments and biochemical testing, and a clinical history was taken. Neurological/neurophysiological assessments comprised calculation of the neuropathy impairment score of the lower limbs (NIS-LL) to quantify signs of neuropathy [19], calculation of the Toronto clinical neuropathy score (TCNS) to assess neuropathy symptoms and signs [20], administration of the Douleur Neuropathique en 4 (DN4) questionnaire to assess for the presence of neuropathic pain [21], and use of the neuropathic pain symptom inventory (NPSI) to evaluate symptoms of neuropathic pain [22]. Nerve conduction studies were performed at controlled skin temperature (31°C) and room temperature (24°C) using an electrophysiological system (Medelec; Synergy Oxford Instruments, Oxford, UK). The following peripheral nerve attributes were measured: (1) sural sensory nerve action potential; (2) common peroneal motor nerve distal latency, compound muscle action potential, and conduction velocity; and (3) tibial motor nerve distal latency.

An established neuropathy composite score, the NIS-LL plus seven neurophysiological tests (NIS-LL+7), was derived from transformed percentile points of abnormalities in nerve conduction studies, vibration detection thresholds acquired from the dorsal aspect of the right foot using computer-assisted evaluation (CASE IV; WR Medical Electronics, Stillwater, MN, USA) using standard techniques [23], and heart rate variability with deep breathing (obtained using a computer-assisted technique according to O’Brien’s protocol [24]), and calculated in accordance with the criteria proposed by Dyck et al [25].

German Research Network of Neuropathic Pain (Deutscher Forschungsverbund Neuropathischer Schmerz [DFNS]) quantitative sensory testing (QST) [26] was also performed, where the following sensory sub-modalities were tested: the cold detection threshold (CDT) and warm detection threshold (WDT) (TSA2; Medoc, Ramat Yishai, Israel), the cold pain threshold (CPT) and heat pain threshold (HPT), the mechanical detection threshold (MDT) (using von Frey hairs, Optihair2 set; Marstock Nervtest, Germany) and mechanical pain threshold (MPT) (using a PinPrick stimulator set; Medizintechnische Systeme, Germany), mechanical pain sensitivity (MPS), dynamic mechanical allodynia (DMA) (using a standardised brush, Somedic, Sweden; plus cotton wool tip and cotton wisp), the pressure pain threshold (PPT) (using a pressure gauge device; Wagner Instruments, Greenwich, CT, USA) and the vibration detection threshold (VDT) (using a Rydel–Seiffer tuning fork, 64 Hz, 8/8 scale). As per the DFNS protocol, all modalities were tested using the same technique at the dorsum of the foot, except for the VDT (medial malleolus) and PPT (abductor hallucis muscle). The QST data were *z*-transformed for each parameter, and compared with site, sex- and age specific reference data. Positive *z* scores indicate a gain of function, and negative *z* scores indicate a loss of function. Formal training for the protocol was provided by members of the DFNS group at Bochum Hospital, Germany. Small-fibre sudomotor function was examined using SUDOSCAN (Impeto Medical) to determine the electrochemical skin conductance (ESC) in the feet [27].

Based on these assessments, participants with diabetes were divided into three groups according to the Toronto criteria [28] for the confirmed diagnosis of DPN, and also the Toronto and International Association for the Study of Pain (IASP) criteria for neuropathic pain/painful DPN [29]: (1) confirmed painless DPN indicated by abnormal clinical neurological findings (TCNS > 5) and two or more nerve conduction abnormalities (one of which had to be an attribute of the sural nerve), in the absence of neuropathic pain; (2) confirmed painful DPN, indicated by abnormal clinical neurological findings (TCNS>5) and two or more nerve conduction abnormalities (one of which had to be an attribute of the sural nerve), a DN4 score ≥4, and chronic neuropathic pain for ≥6 months; and (3) no DPN, indicated by no abnormal clinical neurological findings (TCNS <5), no nerve conduction abnormalities, and no evidence of neuropathic pain.

Participants underwent anthropometric measurements (weight, height and calculation of BMI) and assessment of blood pressure. Blood tests were performed to assess serum HbA_1c_ and creatinine level, and urine biochemistry for urinary albumin/creatinine ratio (ACR). We graded diabetic retinopathy severity as 0, representing no diabetic retinopathy; 1, representing background or pre-proliferative diabetic retinopathy; or 2, representing proliferative diabetic retinopathy, using two-field mydriatic digital photography images acquired as part of the NHS Diabetic Eye Screening Programme. Diabetic nephropathy was defined as a urinary ACR of ≥30 mg/mmol for more than two urine samples more than 3 months apart.

The Hospital Anxiety and Depression Scale (HADS) was used to determine the presence of depression (HADS-D) and anxiety (HADS-A), using a cut-off score of >10 [30].

### Magnetic resonance imaging

Participants with diabetes had a finger-prick blood glucose measured before the scan, and did not have a scan if they had experienced hypoglycaemia (blood glucose level <4 mmol/l) within the preceding 24 h. An 11-point numeric rating scale (NRS: 0=no pain, 10=most severe pain ever) was used to record pain severity during the scan in participants with painful DPN, and had not taken analgesic medications for 48 h. All brain imaging parameters were acquired in one visit.

#### Scan acquisition

**Fig. 1:**
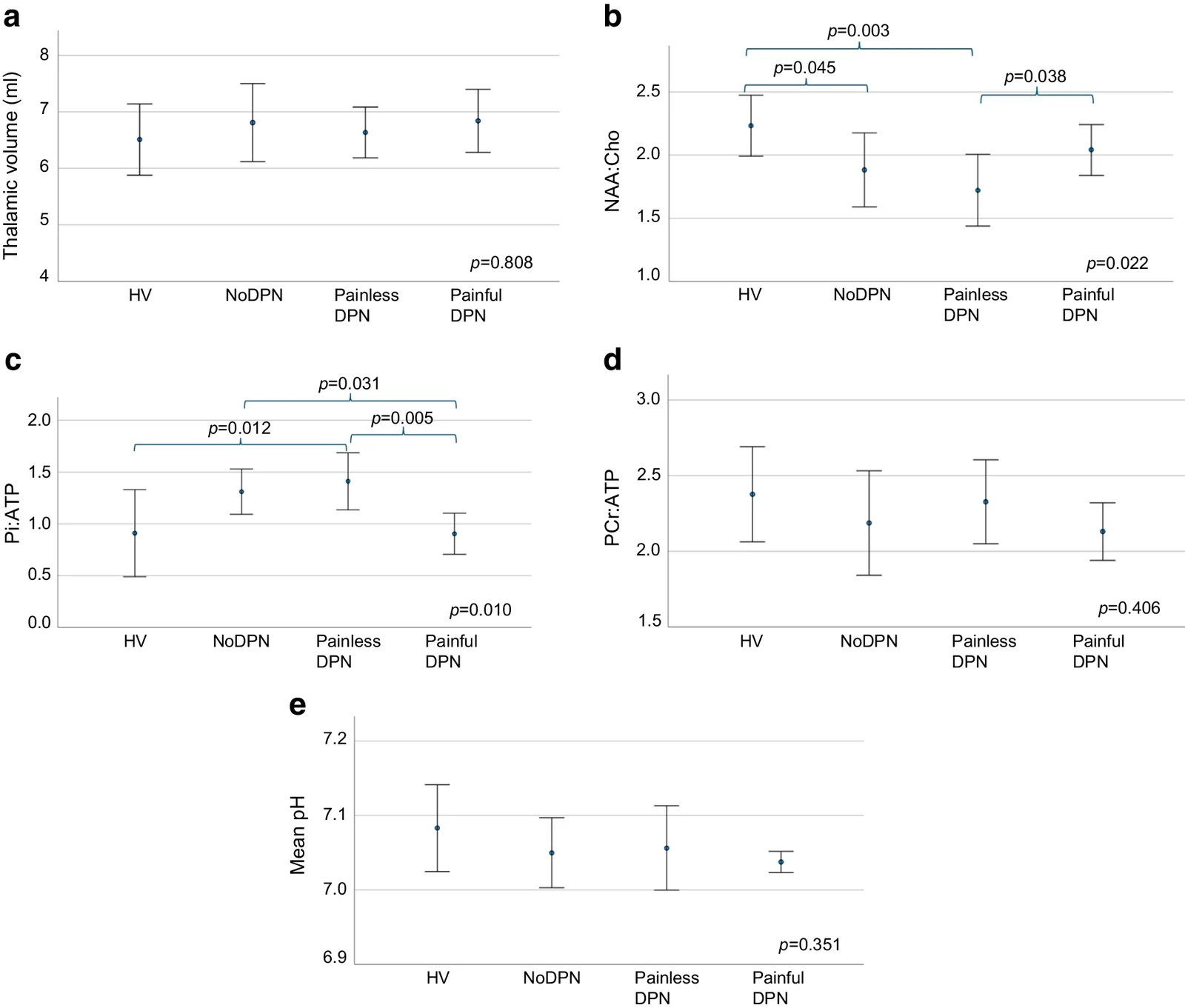
MRI structural and spectroscopy data. (**a**) Structural results, (**b**) ^1^H-MRS results, and (**c**–**e**) ^31^P-MRS results. Groups were compared using ANOVA (*p* values at the bottom of each graph) and post hoc analysis by least-squares difference

Participants underwent cerebral MRI and MRS at 3 Tesla (Ingenia, Philips Healthcare, Best, the Netherlands), using a dual-tuned ^1^H/^31^P head coil (RAPID Biomedical, Germany). A ^1^H survey was run first using the following parameters: fast field echo technique, T1 contrast enhancement, turbo field echo fast imaging mode, flip angle 15°, echo time (TE) 4.6 ms, repetition time (TR) 11 ms, acquisition matrix 256×128, acquisition voxel size 0.98×1.95×10 mm^3^, reconstruction voxel size 0.98×0.98×10 mm^3^, acquisition time 31.4 s. High-resolution, three-dimensional T1-weighted MRI images were then acquired using a magnetisation-prepared rapid gradient echo sequence. Scanning parameters were as follows: fast field echo technique, T1 contrast enhancement, turbo field echo fast imaging mode, flip angle 8°, TE 3.7 ms, TR 8.1 ms, reconstructed voxel size 0.94×0.94×1 mm^3^, reconstruction matrix 256, acquisition time 5 min 28.9 s. A coronal T2-enhanced MRI sequence was run next to aid MRS voxel localisation using the following parameters: multi-spin scan mode, spin-echo technique, flip angle 90°, TE 80 ms, TR 3000 ms, reconstruction matrix 432, reconstruction voxel size 0.53×0.53×4 mm^3^, acquisition time 1 min 54 s. The voxels were placed to encompass the entirety of the thalamus and to correspond between the ^1^H and ^31^P acquisitions (electronic supplementary material [ESM] Fig. 1). ^1^H spectra were acquired using a two-dimensional point resolved spectroscopy sequence (chemical shift imaging-point resolved spectroscopy): TE 288 ms, TR 2000 ms, number of signals averaged=1, auto second-order pencil beam shim, water suppression excitation with 140 Hz bandwidth, acquisition voxel size 25×25×40 mm^3^, reconstruction voxel size 23×23×40 mm^3^, 1024 points in free induction delay, spectral resolution 1.95 Hz/point, field of view 300×300, matrix 12×12, 162 signal acquisitions, acquisition time 5 min 24 s. ^31^P spectra were acquired using a two-dimensional slice-selection image-selected in vivo spectroscopy [31] technique with the following parameters: TE 26 ms, TR 4000 ms, number of signals averaged =2 (288 total signal acquisitions; 144 spatial encodes), auto second-order pencil beam shim, standard broadband decoupling, acquisition voxel size 25×25×40 mm^3^, reconstruction voxel size 23×23×40 mm^3^, 2048 points in free induction delay, spectral resolution 1.465 Hz/point, acquisition time 16 min 24 s. Example spectra are shown in ESM Fig. 1.

#### Structural MRI data analysis

Automated segmentation was performed using FSL-FIRST, an automated model-based segmentation/registration tool carried out using the FMRIB Software Library (FSL, version 6.0.3). FSL-FIRST was used according to published guidelines [32], whereby images were initially registered to the MNI152 standard space template, and registration was visually checked for each participant. FSL-FIRST uses a training data‐based approach and a Bayesian probabilistic model to determine the most probable shape of subcortical structures given the intensities of the T1 image. Surface meshes of the subcortical region of interest (ROI) were converted to boundary-corrected volumetric representations, and boundary correction was automatically generated using the ‘run_first_all’ command line. Successful segmentation of ROI was visually verified, and ROI masks were extracted from the segmented image for subsequent analysis. Differences in left (dominant hemisphere) and right (non-dominant hemisphere) thalamic volume were compared across the study groups. Vertex shape analysis was applied using 5000 permutations, and significance was defined as a *p* value <0.05.

Volumetric segmentation of the thalamus was performed using Freesurfer software (https://surfer.nmr.mgh.harvard.edu). This processing comprises motion correction and averaging [33] of volumetric T1-weighted images, removal of non-brain tissue using a hybrid watershed/surface deformation procedure [34], affine-registered to the Talairach atlas [35], intensity normalisation, tessellation of the grey matter/white matter boundary, automated topology correction [36], and surface deformation following intensity gradients to optimally place the grey/white and grey/cerebrospinal fluid borders at the location where the greatest shift in intensity defines the transition to the other tissue class. Surface-based maps were created from the intensity and continuity information from the entire 3D MRI volume in segmentation and deformation procedures to produce representations of volumetric data for each thalamus [37].

### MRS data analysis

^1^H and ^31^P-MRS signals were processed and fitted using the java-based MR user interface software package jMRUI [38], version 5.2.

#### ^1^H-MRS

The residual water signal was removed using the Hankel–Lanczos squares singular values decomposition (HLSVD) function/feature. Subsequently, spectra were zero-order-phased and the main NAA peak was set to 2.02 ppm. The peaks of interest, consistently identified in the long-echo ^1^H spectra, were those present at 2.02, 3.03 and 3.22 ppm, assigned to NAA, creatine (Cr) and choline (Cho), respectively. After spectra pre-processing, the peaks were quantified using AMARES (Advanced Method for Accurate, Robust and Efficient Spectral fitting) [39] within jMRUI, and the following metabolite ratios were calculated: NAA:Cho, NAA:Cr and Cho:Cr. ^1^H-MRS spectra in which the three main peaks (at 2.02, 3.03 and 3.22 ppm) could not be identified by AMARES, due to poor spectra quality such as poor shim, non-negligible lipid contamination or poor water suppression, have been removed from further analysis.

#### ^31^P-MRS

Spectra were zero- and first-order-phased, and then the phosphocreatine (PCr) peak was set to 0 ppm. Subsequently, the ^31^P-MRS spectra were fitted using the AMARES feature/method within jMRUI, in order to quantify 13 Lorentzian peaks: three for the β-ATP (triplet) and two for α- and γ-ATP (doublets), as well as one peak for each of the following: PCr, glycerophosphocholine, glycerophosphoethanolamine, inorganic phosphate (Pi), phosphoethanolamine and phosphocholine. We applied prior knowledge, such as setting the ratio of the amplitudes of the β-ATP triplet to 1:2:1, while the amplitude ratios for the α-ATP and γ-ATP doublets were set to 1:1. We also optimised and applied soft constraints to the peak linewidths for each component, as follows: the peaks of glycerophosphocholine, glycerophosphoethanolamine, Pi, phosphoethanolamine and phosphocholine were constrained to be less than 25 Hz, while the ATP (β, α and γ) multiplets were constrained to have equal linewidths (the β-ATP peak linewidth ratios were set to 1:1:1; the α-ATP peak ratio was set to 1:1; the γ-ATP peak ratio was set to 1:1) and to be less than 15 Hz. The PCr peak was the only one for which we did not impose any linewidth constraints. Additionally, we imposed a fixed frequency shift between the peaks of β-ATP (16 Hz), α-ATP (18 Hz) and γ-ATP (18 Hz).

### Statistical analysis

By convention, metabolite levels were expressed as ratios. The pre-specified neurometabolite ratios used as outcome measures were NAA:Cho, NAA:Cr and Cho:Cr for ^1^H-MRS and Pi:ATP, PCr:ATP and phosphomonoesters: phosphodiesters (PME:PDE) for ^31^P-MRS data. Moreover, the pH was calculated using the following formula automatically generated within jMRUI: pH=6.75 + log_10_[(δ–3.27)/(5.69–δ)], where δ is the chemical shift frequency difference between pH‐dependent Pi and the pH‐independent reference peak (PCr), measured in ppm. Cramér–Rao bounds for spectroscopy data are shown in ESM Table 1. We present data from the dominant hemisphere as the side we have studied in previous research [11, 14, 40]; left, right and mean thalamic data are presented in the ESM.

Data were tested for normality using the Shapiro–Wilk test. Continuous data are presented as mean ± SD for normally distributed continuous data, median (IQR) for non-normally distributed continuous data, and *n* (%) for categorical data. Group differences in clinical/neurophysiological and MR parameters were compared using one-way ANOVA for normally distributed data, the Kruskal–Wallis rank test for non-normally distributed data, and the χ^2^ test for categorical variables. Post hoc analysis was performed using least-squares difference for normally distributed data and the Mann–Whitney *U* test for non-normally distributed data. Pearsons’ correlation was used for all correlation analyses. All statistical analyses were conducted using SPSS version 28 software.

Based on a sample size calculation performed according to our recent study [13], assuming the outcomes within the groups is normally distributed with an SD of 0.2 and the mean difference in thalamic NAA between groups is 0.35, nine participants were needed in each group to be able identify a group difference with probability 0.90 and alpha of 0.05.

## Results

### Demographic characteristics and clinical assessments

Table 1 displays the demographic characteristics and the results of clinical assessments for study participants. There were no group differences in demographic characteristics such as age and sex, nor clinical parameters such as BMI, systolic blood pressure and serum creatinine, presence of smoking, and current depression or anxiety. As expected, the HbA_1c_ was higher in the diabetes groups compared with HV, but there were no differences in HbA_1c_ between diabetes groups. Moreover, there were no differences between the diabetes groups in terms of the duration of diabetes or the degree of nephropathy and retinopathy. People with painful DPN (*n*=18) were prescribed the following analgesia: four received gabapentin (22%), five received duloxetine (28%), eight received pregabalin (44%), four received tramadol (22%), two received amitriptyline (11%), one received carbamazepine (6%) and two received co-codamol (11%). Two individuals (11%) were receiving no treatment, and five (28%) were receiving combination therapy. There was no difference in finger-prick blood glucose levels between participants taken at the time of the scan.

**Table 1:** Demographic characteristics and clinical assessments for each study cohort

	HV (*n*=11)	No DPN (*n*=9)	Painless DPN (*n*=11)	Painful DPN (*n*=18)	*p* value
Age (years)	65.7 (61–74)	60.0 (59–66)	62.0 (60–69)	61.5 (58.3–66.3)	0.442^a^
Female sex	4 (36%)	5 (56%)	7 (64%)	7 (39%)	0.482^b^
Duration of diabetes (years)		9.7±5.8	16.5±5.6	12.0±7.4	0.065
BMI (kg/m^2^)	26.8±5.5	28.9±4.5	32.3±3.6	30.8±5.1	0.056
HbA_1c_ (mmol/mol)	37.0 (34.0–39.0)	61.0 (47.0–78.5)	56.0 (53.0–73.0)	64.0 (45.8–78.5)	<0.001^a†‡§^
HbA_1c_ (%)	5.5 (5.3–7.5)	7.7 (6.5–9.3)	7.3 (7.0–8.8)	8.0 (6.3–9.3)	
Retinopathy score^c^					0.241^b^
0		5 (56%)	5 (45%)	7 (39%)	
1		4 (44%)	5 (45%)	5 (28%)	
2		0 (0%)	1 (9%)	6 (33%)	
ACR (mg/mmol)		0.64±0.38	1.45±0.89	2.29±3.0	0.070^a^
Creatinine (μmol/l)	70.4±7.4	69.1±9.6	77.0±12.7	71.8±16.7	0.532
Ever smoked	5 (46%)	5 (78%)	6 (55%)	12 (67%)	0.454^b^
Systolic blood pressure (mmHg)	134.2±21.5	130.4±10.7	142.6±17.7	141.3±15.9	0.292
Current depression	0	0	0	3 (17%)	0.138^b^
Current anxiety	0	0	0	3 (17%)	0.317^b^
Blood glucose during MR scan (mmol/l)		9.8 (6.4–12.2)	8.4 (6.2–9.8)	8.6 (5.3–10.2)	0.862^a^

Data are presented as mean ± SD for normally distributed continuous data, median (IQR) for non-normally distributed continuous data, and *n* (%) for categorical data

Groups were compared using ANOVA for continuous data that were normally distributed, the Kruskal–Wallis rank test for continuous data that were not normally distributed (^a^) and the χ^2^ test for categorical data (^b^). A *p* value < 0.05 was considered statistically significant

^†^*p*<0.05 for HV vs no DPN; ^‡^*p*<0.05 for HV vs painless DPN; ^§^*p*<0.05 for HV vs painful DPN

^c^‘0’ represents no diabetic retinopathy; ‘1’ represents background or pre-proliferative diabetic retinopathy; ‘2’ represents proliferative diabetic retinopathy

### Neurological and neurophysiological assessments

Table 2 displays the results of neurological and neurophysiological assessments in study participants. There was a significant group difference in the TCNS (Kruskal–Wallis test, *p*<0.001). The TCNS was significantly higher in painless and painful DPN compared with HV and those with no DPN (Mann–Whitney *U* test, all *p*<0.001). Moreover, as the TCNS has a symptom component, it was significantly higher in painful DPN (13.0±6.3) compared with painless DPN (8.0±12.0, *p*=0.049). The NIS-LL, peroneal amplitude, peroneal velocity, peroneal latency, sural amplitude, tibial latency and ESC were significantly different in DPN groups compared with non-DPN groups (all *p*<0.05). As expected, the DN4 score (6.7±1.5) and total NPSI score (21.4±9.9) were significantly higher in painful DPN compared with all other groups (all *p*<0.001), with no differences among the other groups.

**Table 2:** Neurological and neurophysiological assessments for each study cohort

	HV (*n*=11)	No DPN (*n*=9)	Painless DPN (*n*=11)	Painful DPN (*n*=18)	*p* value
TCNS	0.0 (0.0-0.0)	0.0 (0.0-2.0)	8.0 (5.0-20.5)	13.0 (6.3-21.0)	<0.001^*†‡§¶a^
NIS-LL	0.0 (0.0-0.0)	0.0 (0.0-0.0)	11.0 (5.0-20.5)	14.0 (6.3-21.0)	<0.001^*†§¶a^
Peroneal amplitude (mV)	6.0±2.4	6.0±2.1	1.7±1.8	2.5±1.9	<0.001^*†§¶^
Peroneal velocity (m/s)	46.7±1.7	47.2±4.6	33.8±14.0	37.7±5.0	<0.001^*†§¶^
Peroneal latency (msec)	4.9 (4.6-5.3)	4.2 (4.1-4.5)	6.5 (4.7-8.6)	5.6 (4.8-6.1)	0.02^*†‡a^
Tibial latency (msec)	4.6 (4.0-4.9)	4.5 (4.3-4.7)	5.4 (4.7-7.1)	6.1 (4.7-7.9)	0.001^*§¶a^
Sural amplitude (mV)	13.6 (11.1-18.1)	12.5 (9.2-13.2)	1.8 (0.9-4.3)	0.6 (0.2-7.9)	<0.001^*†§¶a^
NIS-LL+7	0.0 (0.0-1.0)	0.0 (0.0-0.0)	18.0 (11.5-29.0)	21.0 (14.0-27.3)	<0.001^*†§¶a^
ESC (μS)	79.0 (67.8-84.3)	76.0 (64.0-81.0)	65.0 (46.5-74.0)	58.0 (33.8-70.0)	0.001^*§¶a^

Data are presented as mean ± SD for normally distributed continuous data, and median (IQR) for non-normally distributed continuous data

Groups were compared using ANOVA for continuous data that were normally distributed, and the Kruskal–Wallis rank test for continuous data that were not normally distributed (^a^). A *p* value < 0.05 was considered statistically significant

^*^p<0.05 for HV vs painless DPN; ^†^*p*<0.05 for no DPN vs painless DPN;^‡^*p*<0.05 for painless DPN vs painful DPN; ^§^*p*<0.05 for HV vs painful DPN; ^¶^*p*<0.05 for no DPN vs painful DPN

The CDT, WDT, thermal sensory limen, MPT, MPS, MDT and VDT were all significantly lower in the DPN groups compared with the non-DPN groups (all *p*<0.05; ESM Table 2), with no differences between painful and painless DPN. The HPT was significantly lower in painful DPN (−1.38±0.58, ANOVA, *p*=0.005) compared with HV (0.49±1.52, *p*=0.002; 95% CI 0.54, 2.33) and no DPN (−0.09±1.24, *p*=0.009; 95% CI −2.25, −0.34) and was lower than in painless DPN (−1.10±1.32, *p*=0.024; 95% CI −2.14, −0.16).

### MR analysis results

Table 3 and Fig. 1 show the MR analysis results for the dominant hemisphere, ESM Table 3 and ESM Fig. 2 show MR analysis for non-dominant and mean thalamic data. On structural brain analysis, there was no difference in thalamic volume on either assessment modality using Freesurfer (Table 3 and Fig. 1) or FSL-FIRST. Eleven participants were excluded from the ^1^H-MRS analysis, due to inadequate spectra being obtained because of participant movement. Therefore, for the ^1^H-MRS analysis, a total of 38 participants were included (nine HV, seven with no DPN, eight with painless DPN and 14 with painful DPN). There was a significant group effect for the NAA:Cho ratio (ANOVA, *p*=0.022), which was significantly higher in HV (2.23±0.32) compared to those with no DPN (1.88±0.32, *p*=0.045; 95% CI 0.01, 0.69) or painless DPN (1.72±0.34, *p*=0.003; 95% CI 0.18, 0.84), and also higher in those with painful DPN (2.04±0.35) compared with painless DPN (*p*=0.038; 95% CI −0.02, 0.62).

**Table 3:** Thalamic structural, ^1^H- and ^31^P-MRS results

	HV (*n*=11)	No DPN (*n*=9)	Painless DPN (*n*=11)	Painful DPN (*n*=18)	ANOVA
Volume (ml)	6.51±0.93	6.81±0.90	6.63±0.67	6.84±1.12	0.808
NAA:Cho^a^	2.23±0.32	1.88±0.32	1.72±0.34	2.04±0.35	0.022^§^*^‡^
NAA:Cr^a^	2.37±0.69	2.38±0.84	2.08±0.62	2.40±0.64	0.721
Cho:Cr^a^	1.15±0.28	1.37±0.56	1.65±1.05	1.33±0.38	0.411
Pi:ATP^b^	0.91±0.63	1.31±0.28	1.41±0.41	0.90±0.40	0.010*^†‡^
PCr:ATP^b^	2.38±0.47	2.19±0.45	2.33±0.41	2.13±0.38	0.406
PME:PDE^b^	0.96±0.47	1.17±0.39	1.00±0.46	1.05±0.49	0.768
pH^b^	7.08±0.08	7.05±0.06	7.06±0.08	7.04±0.03	0.351

Data are presented as mean ± SD

Groups were compared using ANOVA. A *p* value < 0.05 was considered statistically significant

^a^Data are ^1^H-MRS metabolite ratios

^b^Data are ^31^P-MRS metabolite ratios, expressed relative to ATP

^*^p<0.05 for HV vs painless DPN; †*p*<0.05 for HV vs painful DPN; ‡*p*<0.05 for no DPN vs painless DPN; §*p*<0.05 for painless DPN vs painful DPN

For the ^31^P-MRS data, the mean of the α-ATP and γ-ATP peaks was used to calculate total ATP; the β-ATP peak was not included as it was not identified in all spectra. There was a significant group effect for the Pi:ATP ratio (ANOVA, *p*=0.010) (Fig. 1). The Pi:ATP ratio was significantly lower in HV (0.91±0.63) compared to those with painless DPN (1.41±0.41, *p*=0.012; 95% CI −0.88, −0.12) and there was a trend towards it being lower than those with no DPN (1.31±0.28, *p*=0.052). The Pi:ATP ratio was also significantly lower in painful DPN (0.90±0.40) compared to those with no DPN (*p*=0.031; 95% CI −0.12, 0.88) or painless DPN (*p*=0.005; 95% CI −0.85, −0.16). There was no group difference in thalamic PCr:ATP (*p*=0.406), PME:PDE (*p*=0.768) or pH (*p*=0.351) (Fig. 1 and Table 3).

### Correlation analysis

Thalamic volume correlated with age (Pearson’s *r* = −0.351, *p*=0.011) and pH (*r* = 0.312, *p*=0.027). ESM Tables 4–6 show the correlation analysis results in all groups for NAA:Cho, Pi:ATP and PCr:ATP vs clinical/neurological variables. NAA:Cho correlated with BMI (*r* =−0.444, *p*=0.005), peroneal conduction velocity (*r* = 0.438, *p*=0.014) and PPT (*r* = −0.336, *p*=0.039). Pi:ATP correlated with BMI (*r* = 0.346, *p*=0.015) and PCr:ATP correlated with HbA_1c_ (*r* = −0.310, *p*=0.030).

**Fig. 2:**
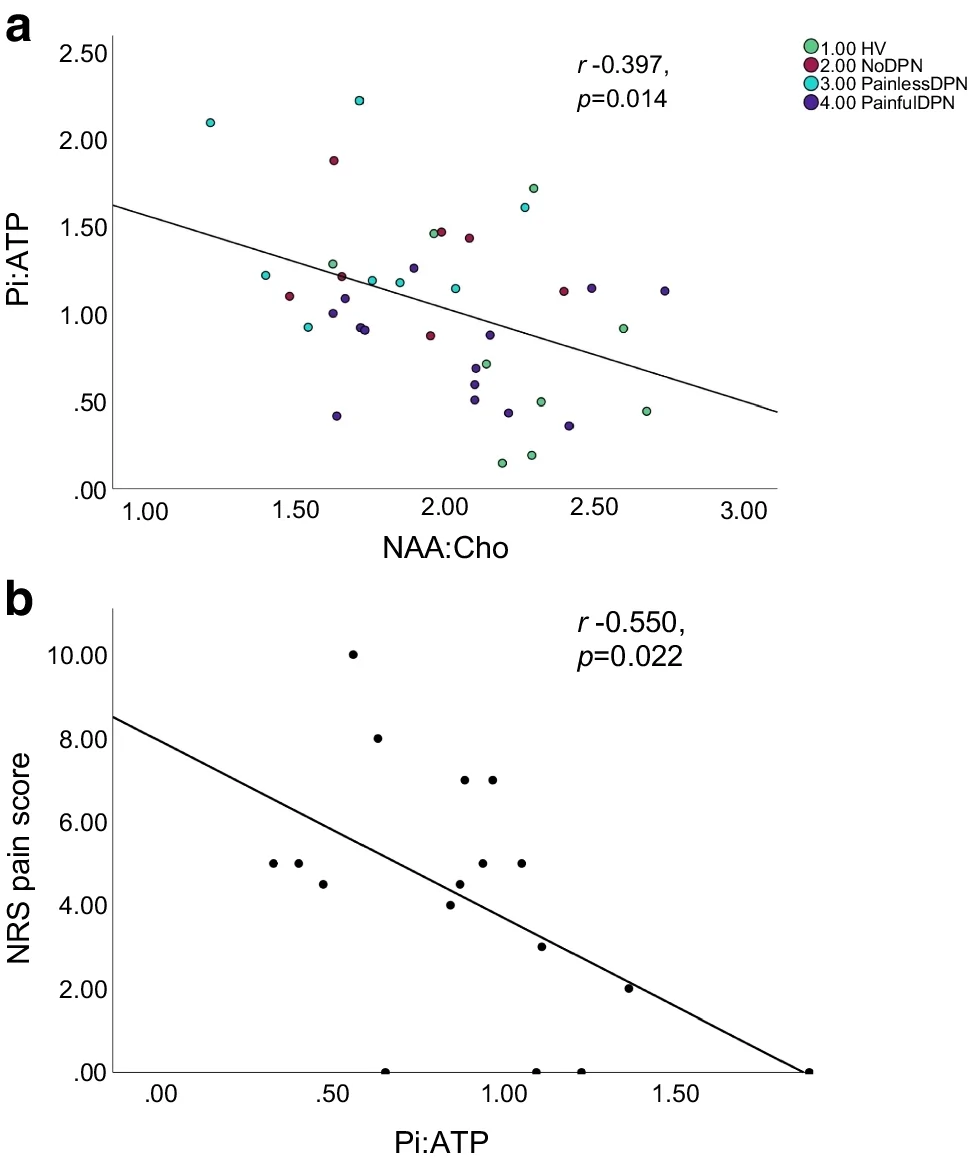
Pearson’s correlations at the thalamus. (**a**) Correlation between Pi:ATP and NAA:Cho. (**b**) Correlation between the NRS pain score and Pi:ATP

When looking exclusively at measures of neuropathic pain within the painful DPN group, the NRS pain score during the MR correlated with thalamic Pi:ATP (*r* = −0.550, *p*=0.022; Fig. 2) and PCr:ATP correlated with paroxysmal pain in the NPSI (*r =* −0.514, *p*=0.029). We then correlated ^1^H- and ^31^P-MRS measures (ESM Table 7). The NAA:Cho ratio significantly correlated with Pi:ATP at the thalamus (*r* = −0.397, *p*=0.014; Fig. 2).

## Discussion

This study combined thalamic structural imaging with ^31^P-MRS and ^1^H-MRS neuroimaging in the same DPN cohort. Despite no differences in thalamic volume, there was evidence of ^1^H- and ^31^P-MRS neurometabolite alterations among the groups. The NAA:Cho ratio, a marker of neuronal function, was lowest in individuals with painless DPN, while the levels in those with painful DPN were equivalent to those in HV. Furthermore, Pi:ATP levels, a marker of thalamic high-energy phosphate metabolism and bioenergetic state, were found to be highest in painless DPN, suggesting altered bioenergetics, potentially consistent with impaired mitochondrial oxidative phosphorylation in the thalamus, while the levels in those with painful DPN were again similar to those in HV.

NAA is a highly abundant molecule in the central nervous system, with key involvement in neuronal osmoregulation, myelin lipid synthesis and assisting nitrogen removal from neurons. Quantification of cerebral NAA using ^1^H-MRS is a reproducible, robust and widely used marker of numerous neurological disorders, with a low level being suggestive of reduced neuronal viability and/or function [41]. Our group and others have previously demonstrated a reduction in NAA in the thalamus in people with type 1 diabetes and painless DPN compared to people with diabetes but without DPN and healthy volunteers [14, 15, 42]. In the present cohort of people with type 2 diabetes, we found a reduction in NAA in the no DPN and painless DPN groups, with the lowest levels in those with painless DPN. Thus, these findings are consistent with our previous research, which demonstrated thalamic neuronal dysfunction in painless DPN in both people with type 1 diabetes and those with type 2 diabetes. The mechanisms underlying thalamic neuronal dysfunction in painless DPN remain unclear, but may reflect loss of afferent input to the thalamus, causing a dying back of central nervous system neurons. Alternatively, there may be primary thalamic neuronal injury, resulting in impaired pain perception, but longitudinal studies are required to clarify the aetiology of this further.

In this study, ^31^P-MRS was performed for the first time in a cohort with DPN, to determine thalamic bioenergetic status (Pi:ATP), cellular bioenergetics (PCr:ATP), membrane turnover (PME:PDE) and pH. The most significant finding was an increase in Pi:ATP in individuals with painless DPN, indicating altered high-energy phosphate metabolism, consistent with a disturbance in oxidative phosphorylation-related bioenergetics [43, 44]. Thus, these results suggest altered thalamic bioenergetics in those with painless DPN and to a lesser extent in those with diabetes but without DPN, similar to the pattern observed for NAA:Cho. Importantly, we found a clear relationship between Pi:ATP and NAA:Cho ratios, suggesting a link between thalamic mitochondrial and neuronal function. Although oxidative stress and mitochondrial dysfunction are well recognised peripheral pathophysiological contributors to DPN [45], and have been implicated in cerebral complications of diabetes [46], it remains unclear whether altered thalamic bioenergetics contributes to neuronal dysfunction or vice versa. Determining the causal relationship will require prospective studies.

The results also suggest that relative preservation (or potential upregulation) of thalamic bioenergetic and neuronal profiles, rather than impairment, appears to be a central mechanism of painful DPN. These findings are consistent with other recent MRS research studies that found preserved NAA levels and neurotransmitter levels (GABA) in painful DPN compared with painless DPN [11, 14]. Moreover, rodent models of painful DPN show the presence of hyperexcitable thalamic neurons in response to stimuli and enhanced spontaneous activity [47]. Recently, we also found that, on withdrawal of analgesia in individuals with painful DPN, there is an increased engagement in the thalamo-cortical pathway [40]. These findings are consistent with thalamic hyperperfusion in individuals with painful DPN compared with the sluggish blood flow in those with painless DPN [17]. The correlation between Pi:ATP and the severity of neuropathic pain further suggests a role for mitochondrial function in the central mechanisms of painful DPN.

The study’s main strength lies in the simultaneous application of multimodal MR techniques in the same participants, reducing potential confounding factors related to cohort differences and scan parameters. Moreover, MRS is a robust and reproducible technique, and integrating modalities within a single cohort strengthens interpretability [41]. Additional strengths include the inclusion of four well-characterised participant groups, comprising healthy volunteers and individuals with type 2 diabetes with and without DPN, allowing the effects of diabetes and DPN on the thalamus to be distinguished. Moreover, the participants underwent detailed clinical phenotyping, which demonstrated that there were no significant group differences in important demographic, clinical and metabolic factors, with no differences in objective measures of neuropathy between individuals with painless DPN and those with painful DPN. However, this study has some limitations. First, while comparable to those in prior neuroimaging studies [13, 44], the sample size remains modest, particularly for subgroup and correlation analyses (including sex analyses), which may limit statistical power and increase the risk of type I and II errors. Although groups were broadly well matched, residual confounding from factors such as BMI and HbA_1c_ cannot be excluded, as the sample size limited adjusted analyses. Given the cross-sectional design, modest sample size and multimodal imaging design, these findings should be considered exploratory, and causation requires confirmation in larger prospective studies.

This is the first study to apply dual ^1^H- and ^31^P-MRS in people with DPN. Together with previous work, these findings support thalamic neuronal hyperactivity in individuals with painful DPN, with a preservation of bioenergetic and neuronal profiles. These findings may have clinical relevance, as ^31^P-MRS metabolites may potentially be used as objective biomarkers of painful DPN or neuropathic pain. Prospective research is necessary to determine the mechanisms underlying thalamic neuronal and bioenergetic dysfunction in painless DPN, and whether these mechanisms could represent targets for disease-modifying therapies.

## Supplementary Information

Below is the link to the electronic supplementary material.

ESM (PDF 372 KB)

## Data Availability

The de-identified data that support the findings of this study are available from the corresponding author upon reasonable request for up to 5 years following publication. Data will be shared for non-commercial research purposes, subject to institutional approval, participant consent, and applicable data protection regulations.

## Abbreviations

ACR: Albumin/creatinine ratio
AMARES: Advanced Method for Accurate, Robust and Efficient Spectral fitting
CDT: Cold detection threshold
Cho: Choline
CPT: Cold pain threshold
Cr: Creatine
DFNS: German Research Network of Neuropathic Pain/Deutscher Forschungsverbund Neuropathischer Schmerz
DN4: Douleur Neuropathique en 4
DPN: Diabetic peripheral neuropathy
ESC: Electrochemical skin conductance
GABA: γ-Aminobutyric acid
HADS: Hospital Anxiety and Depression Scale
HPT: Heat pain threshold
HV: Healthy volunteers
IASP: International Association for the Study of Pain
MDT: Mechanical detection threshold
MPS: Mechanical pain sensitivity
MPT: Mechanical pain threshold
MRS: Magnetic resonance spectroscopy
NAA: *N*-Acetylaspartate
NIS-LL: Neuropathy impairment score of the lower limbs
NIS-LL+7: NIS-LL plus seven neurophysiological tests
NPSI: Neuropathic pain symptom inventory
NRS: Numeric rating scale
PCr: Phosphocreatine
PDE: Phosphodiesters
Pi: Inorganic phosphate
PME: Phosphomonoesters
PPT: Pressure pain threshold
QST: Quantitative sensory testing
ROI: Region of interest
TCNS: Toronto clinical neuropathy score
TE: Echo time
TR: Repetition time
VDT: Vibration detection threshold
WDT: Warm detection threshold
