# Supplementary material for: Thalamic neuronal and bioenergetic function in painful diabetic peripheral neuropathy: a dual magnetic resonance spectroscopy study
Source: Diabetologia. 2026 Sep 10;69(12):3667–78. doi: 10.1007/s00125-026-06846-8 (PMC13714020; doi:10.1007/s00125-026-06846-8)
Supplement: Supplementary file 1 [file 125_2026_6846_MOESM1_ESM.pdf]

**ESM Table 1. Cramér-Rao bound for $^{1}H$- and $^{31}P$-MRS analysis.**

| | Right | Left |
|---|---|---|
| NAA | 3.9 ± 3.0 | 4.1 ± 4.1 |
| Cr | 10.8 ± 7.8 | 11.9 ± 14.0 |
| Cho | 9.5 ± 3.7 | 8.3 ± 5.7 |
| Β-ATP | 38.4 ± 16.2 | 36.8 ± 25.1 |
| α-ATP | 18.5 ± 3.6 | 19.1 ± 4.2 |
| γ-ATP | 22.4 ± 5.0 | 21.6 ± 3.9 |
| PCr | 11.2 ± 2.4 | 10.9 ± 2.1 |
| Pi | 45.4 ± 32.5 | 41.2 ± 15.2 |
| PME | 70.0 ± 43.9 | 72.9 ± 49.3 |
| PDE | 41.4 ± 18.0 | 44.8 ± 26.0 |

ATP, adenosine triphosphate; Cho, choline; Cr; creatine; NAA, N-acetylaspartae; PCr, phosphocreatine; PDE, phosphodiesters; PME, phosphomonoesters; Pi, inorganic phosphate.

**ESM Table 2. German pain research network (DFNS) results for each study cohort.**

| | HV (n=11) | No-DPN (n=9) | Painless-DPN (n=11) | Painful-DPN (n=18) | ANOVA |
|---|---|---|---|---|---|
| CDT (z-score) | -0.61 ± 1.48 | -0.47 ± 0.93 | -2.32 ± 1.25 | -2.70 ± 0.65 | **<0.001**$^{*\dagger\S\Vert}$ |
| WDT (z-score) | -0.59 ± 0.93 | -0.32 ± 0.94 | -1.86 ± 0.98 | -1.98 ± 0.52 | **<0.001**$^{*\dagger\S\Vert}$ |
| TSL (z-score) | -1.04 ± 1.38 | -0.60 ± 0.88 | -2.32 ± 1.31 | -2.41 ± 0.63 | **<0.001**$^{*\dagger\S\Vert}$ |
| CPT (z-score) | -0.86 ± 0.45 | -0.96 ± 0.25 | -0.88 ± 0.44 | -0.89 ± 0.27 | 0.932 |
| HPT (z-score) | 0.49 ± 1.52 | -0.09 ± 1.24 | -1.10 ± 1.32 | -1.38 ± 0.58 | **0.005**$^{*\dagger\S\Vert}$ |
| PPT (z-score) | 0.88 ± 0.84 | 1.22 ± 0.98 | 1.01 ± 2.04 | 0.58 ± 2.01 | 0.797 |
| MPT (z-score) | 1.55 ± 2.08 | 1.43 ± 1.64 | -2.62 ± 1.00 | -1.70 ± 1.76 | **<0.001**$^{*\dagger\S\Vert}$ |
| MPS (z-score) | -0.38 ± 1.38 | -0.60 ± 1.47 | -1.79 ± 0.55 | -1.62 ± 0.54 | **0.002**$^{*\dagger\S\Vert}$ |
| MDT (z-score) | 1.06 ± 1.39 | 0.94 ± 1.22 | -2.51 ± 1.76 | -2.37 ± 1.41 | **<0.001**$^{*\dagger\S\Vert}$ |
| VDT (z-score) | -0.00 ± 1.30 | -0.22 ± 1.13 | -2.47 ± 2.42 | -2.78 ± 1.73 | **<0.001**$^{*\dagger\S\Vert}$ |
| DMA present (%) | 0 | 0 | 0 | 0 | n/a |

Groups were compared using ANOVA for continuous data. Boldface denotes $p<0.05$. CDT, cold detection threshold; CPT, cold pain threshold; DMA, dynamic mechanical allodynia; HDT, heat detection threshold; HPT, heat pain threshold; MDT, mechanical detection threshold; MPS, mechanical pain sensitivity; MPT, mechanical pain threshold; PPT, pressure pain threshold; VDT, vibration detection threshold. *p < 0.05 HV vs Painless-DPN; † p <0.05 No-DPN vs Painless-DPN; ‡ p <0.05 Painless-DPN vs Painful-DPN; § p <0.05 HV vs Painful-DPN; || p <0.05 No-DPN vs Painful-DPN.

**ESM Table 3. MR analysis results for left, right and mean (total) thalamic structural, 1H- and 31P-MRS results.**

| | HV (n=11) | No-DPN (n=9) | Painless-DPN (n=11) | Painful-DPN (n=18) | ANOVA |
|---|---|---|---|---|---|
| Left Volume, mL | 6.51 ± 0.93 | 6.81 ± 0.90 | 6.63 ± 0.67 | 6.84 ± 1.12 | 0.808 |
| Left NAA:Cho[¶] | 2.23 ± 0.32 | 1.88 ± 0.32 | 1.72 ± 0.34 | 2.04 ± 0.35 | **0.022**[§*‡] |
| Left NAA:Cr[¶] | 2.37 ± 0.69 | 2.38 ± 0.84 | 2.08 ± 0.62 | 2.40 ± 0.64 | 0.721 |
| Left Cho:Cr[¶] | 1.15 ± 0.28 | 1.37 ± 0.56 | 1.65 ± 1.05 | 1.33 ± 0.38 | 0.411 |
| Left Pi:ATP | 0.91 ± 0.63 | 1.31 ± 0.28 | 1.41 ± 0.41 | 0.90 ± 0.40 | **0.010***[†‡] |
| Left PCr:ATP | 2.38 ± 0.47 | 2.19 ± 0.45 | 2.33 ± 0.41 | 2.13 ± 0.38 | 0.406 |
| Left PME:PDE | 0.96 ± 0.47 | 1.17 ± 0.39 | 1.00 ± 0.46 | 1.05 ± 0.49 | 0.768 |
| Left pH | 7.08 ± 0.08 | 7.05 ± 0.06 | 7.06 ± 0.08 | 7.04 ± 0.03 | 0.351 |
| Right Volume, mL | 5.89 ± 0.74 | 5.97 ± 0.54 | 5.73 ± 0.58 | 5.85 ± 0.68 | 0.873 |
| Right NAA:Cho[¶] | 2.00 ± 0.24 | 1.93 ± 0.31 | 1.80 ± 2.74 | 2.00 ± 0.32 | 0.515 |
| Right NAA:Cr[¶] | 2.57 ± 0.71 | 2.45 ± 0.59 | 1.99 ± 0.35 | 2.72 ± 1.01 | 0.219 |
| Right Cho:Cr[¶] | 1.18 ± 0.33 | 1.30 ± 0.61 | 1.19 ± 0.37 | 1.23 ± 0.29 | 0.941 |
| Right Pi:ATP | 0.85 ± 0.39 | 1.10 ± 0.59 | 1.25 ± 0.65 | 0.89 ± 0.52 | 0.241 |
| Right PCr:ATP | 2.28 ± 0.24 | 2.37 ± 0.35 | 2.13 ± 0.34 | 2.04 ± 0.27 | **0.036**[†‖] |
| Right PME:PDE | 1.15 ± 0.98 | 0.87 ± 0.31 | 0.96 ± 0.25 | 1.04 ± 0.59 | 0.759 |
| Right pH | 7.12 ± 0.09 | 7.05 ± 0.05 | 7.02 ± 0.03 | 7.05 ± 0.10 | 0.051 |
| Mean Volume, mL | 6.20 ± 0.82 | 6.39 ± 0.69 | 6.18 ± 0.47 | 6.34 ± 0.86 | 0.890 |
| Mean NAA:Cho[¶] | 2.11 ± 0.26 | 1.90 ± .031 | 1.76 ± 0.24 | 2.00 ± 0.30 | 0.081 |
| Mean NAA:Cr[¶] | 2.47 ± 0.65 | 2.41 ± 0.71 | 2.07 ± 0.31 | 2.56 ± 0.73 | 0.390 |
| Mean Cho:Cr[¶] | 1.17 ± 0.27 | 1.34 ± 0.58 | 1.42 ± 0.61 | 1.42 ± 0.61 | 0.678 |
| Mean Pi:ATP | 0.88 ± 0.41 | 1.21 ± 0.30 | 1.33 ± 0.46 | 0.90 ± 0.36 | **0.012***[‡] |
| Mean PCr:ATP | 2.33 ± 0.30 | 2.28 ± 0.37 | 2.23 ± 0.34 | 2.09 ± 0.26 | 0.197 |
| Mean PME:PDE | 1.06 ± 0.59 | 1.02 ± 0.23 | 0.98 ± 0.30 | 1.04 ± 0.45 | 0.972 |
| Mean pH | 7.10 ± 0.07 | 7.05 ± 0.05 | 7.04 ± 0.04 | 7.04 ± 0.06 | **0.050***[‖] |

Groups were compared using ANOVA for continuous data. Boldface denotes p<0.05. ATP, adenosine triphosphate; Cho, choline; Cr; creatine; NAA, N-acetyl aspartate; PCr, phosphocreatine; Pi, inorganic phosphate. * p<0.05 HV vs Painless-DPN; † p <0.05 No-DPN vs Painful-DPN; ‡ p <0.05 Painless-DPN vs Painful-DPN; § p <0.05 HV vs No-DPN; || p <0.05 HV vs Painful-DPN. ¶ 11 people excluded from analysis due to inadequate $^{1}$H-spectra (HV n=2, No-DPN n=2, Painless-DPN n=3, Painful-DPN n=4).

**ESM Table 4. Correlation analysis of NAA:Cho and clinical/neurological variables.**

| | Right (r, p-value) | Left (r, p-value) | Mean (r, p-value) |
|---|---|---|---|
| Age, years | -2.06, 0.214 | -0.26, 0.877 | -0.122, 0.542 |
| Duration of Diabetes, years | 0.085, 0.662 | -0.276, 0.147 | 0.122, 0.528 |
| Body Mass Index, kg/m$^2$ | -0.294, 0.073 | **-0.444, 0.005** | **-0.416, 0.009** |
| Systolic blood pressure, mmHg | 0.076, 0.649 | 0.076, 0.651 | 0.084, 0.618 |
| HbA1c, mmol/mol | 0.254, 0.124 | -0.131, 0.432 | 0.042, 0.803 |
| Total Cholesterol, mmol/l | 0.018, 0.915 | 0.009, 0.957 | 0.014, 0.933 |
| ACR, mg/mmol | 0.130, 0.546 | -0.148, 0.490 | -0.021, 0.924 |
| Creatinine, µmol/l | 0.021, 0.901 | -0.094, 0.575 | -0.048, 0.776 |
| TCNS | 0.076, 0.650 | -0.105, 0.531 | -0.028, 0.868 |
| NIS-LL | 0.060, 0.721 | -0.227, 0.170 | -0.111, 0.506 |
| Peroneal CMAP (mV) | 0.095, 0.598 | 0.253, 0.155 | 0.0200, 0.265 |
| Peroneal MNCV (m/s) | 0.169, 0.364 | **0.438, 0.014** | 0.348, 0.055 |
| Peroneal MNDL (msec) | -0.212, 0.236 | -0.331, 0.060 | -0.300, 0.089 |
| Tibial MNDL (msec) | 0.104, 0.546 | -0.103, 0.548 | -0.013, 0.940 |
| Sural SNAP (mV) | 0.006, 0.977 | -0.025, 0.898 | -0.012, 0.950 |
| NIS(LL)+7 | 0.147, 0.385 | -0.223, 0.184 | -0.067, 0.693 |
| ESC (µS) | 0.027, 0.875 | 0.224, 0.189 | 0.151, 0.378 |
| CDT, z-score | -0.217, 0.191 | -0.050, 0.764 | -0.136, 0.417 |
| WDT, z-score | -0.097, 0.564 | 0.123, 0.462 | 0.029, 0.862 |
| TSL, z-score | -0.073, 0.662 | 0.102, 0.541 | 0.028, 0.869 |
| CPT, z-score | -0.224, -0.159 | -0.87, 0.602 | -0.162, 0.331 |
| HPT, z-score | -0.159, 0.341 | 0.023, 0.893 | -0.063, 0.709 |
| PPT, z-score | **-0.361, 0.026** | **-0.336, 0.039** | **-0.381, 0.018** |
| MPT, z-score | -0.274, 0.096 | 0.082, 0.623 | -0.081, 0.627 |
| MPS, z-score | -0.036, 0.828 | 0.110, 0.509 | -0.050, 0.763 |
| WUR, z-score | -0.008, 0.976 | -0.127, 0.638 | -0.32, 0.903 |
| MDT, z-score | 0.055, 0.742 | 0.258, 0.117 | 0.186, 0.264 |
| VDT, z-score | 0.144, 0.390 | 0.313, 0.056 | 0.262, 0.112 |

CDT, cold detection threshold; CMAP, compound muscle action potential; CPT, cold pain threshold; DMA, dynamic mechanical allodynia; ESC, electrochemical skin conductance; HDT, heat detection threshold; HPT, heat pain threshold; MDT, mechanical detection threshold; MNCV, motor nerve conduction velocity; MNDL, motor nerve distal latency; MPS, mechanical pain sensitivity; MPT, mechanical pain threshold; NIS-LL, Neuropathy Impairment score of the lower limb; NIS(LL)+7, Neuropathy Impairment score of the lower limb plus 7 neurophysiological tests; PPT, pressure pain threshold; SNAP, sensory nerve action potential; TCNS, Toronto clinical neuropathy score; VDT, vibration detection threshold.

**ESM Table 5. Correlation analysis of PCr:ATP and clinical/neurological variables.**

| | Right (r, p-value) | Left (r, p-value) | Mean (r, p-value) |
|---|---|---|---|
| Age, years | -0.032, 0.826 | 0.190, 0.191 | 0.111, 0.448 |
| Duration of Diabetes, years | 0.005, 0.975 | 0.035, 0.834 | 0.025, 0.880 |
| Body Mass Index, kg/m$^2$ | -0.105, 0.475 | -0.047, 0.750 | -0.83, 0.571 |
| Systolic blood pressure, mmHg | -0.247, 0.087 | -0.041, 0.779 | -0.150, 0.304 |
| HbA1c, mmol/mol | -0.233, 0.107 | **-0.310, 0.030** | **-0.322, 0.024** |
| Total Cholesterol, mmol/l | -0.002, 0.988 | -0.051, 0.729 | -0.035, 0.812 |
| ACR, mg/mmol | -0.220, 0.218 | 0.046, 0.798 | -0.82, 0.649 |
| Creatinine, µmol/l | -0.105, 0.472 | 0.019, 0.896 | -0.039, 0.788 |
| TCNS | **-0.424, 0.002** | -0.115, 0.430 | **-0.287, 0.046** |
| NIS-LL | **-0.380, 0.007** | -0.048, 0.746 | -0.220, 0.129 |
| Peroneal CMAP (mV) | **-0.506, <0.001** | 0.147, 0.340 | **-0.340, 0.024** |
| Peroneal MNCV (m/s) | 0.112, 0.481 | -0.066, 0.676 | 0.009, 0.954 |
| Peroneal MNDL (msec) | 0.017, 0.910 | 0.246, 0.107 | 0.175, 0.257 |
| Tibial MNDL (msec) | **-0.535, <0.001** | -0.157, 0.291 | **-0.368, 0.011** |
| Sural SNAP (mV) | **-0.353, 0.030** | 0.066, 0.694 | 0.211, 0.205 |
| NIS(LL)+7 | **-0.470, <0.001** | -0.059, 0.692 | -0.270, 0.063 |
| ESC (µS) | 0.264, 0.073 | 0.137, 0.358 | 0.221, 0.136 |
| CDT, z-score | **0.374, 0.009** | 0.042, 0.776 | 0.215, 0.143 |
| WDT, z-score | **0.316, 0.029** | 0.024, 0.873 | 0.173, 0.239 |
| TSL, z-score | 0.172, 0.242 | -0.083, 0.575 | 0.031, 0.835 |
| CPT, z-score | 0.084, 0.571 | -0.073, 0.621 | -0.007, 0.963 |
| HPT, z-score | 0.257, 0.078 | 0.042, 0.779 | 0.156, 0.290 |
| PPT, z-score | 0.143, 0.331 | 0.269, 0.065 | 0.250, 0.086 |
| MPT, z-score | 0**.452, 0.001** | 0.115, 0.435 | **0.302, 0.037** |
| MPS, z-score | 0.235, 0.108 | 0.115, 0.437 | 0.194, 0.187 |
| MDT, z-score | **0.404, 0.004** | 0.088, 0.553 | 0.260, 0.074 |
| VDT, z-score | 0.181, 0.217 | -0.031, 0.836 | 0.070, 0.636 |

CDT, cold detection threshold; CMAP, compound muscle action potential; CPT, cold pain threshold; DMA, dynamic mechanical allodynia; ESC, electrochemical skin conductance; HDT, heat detection threshold; HPT, heat pain threshold; MDT, mechanical detection threshold; MNCV, motor nerve conduction velocity; MNDL, motor nerve distal latency; MPS, mechanical pain sensitivity; MPT, mechanical pain threshold; NIS-LL, Neuropathy Impairment score of the lower limb; NIS(LL)+7, Neuropathy Impairment score of the lower limb plus 7 neurophysiological tests; PPT, pressure pain threshold; SNAP, sensory nerve action potential; TCNS, Toronto clinical neuropathy score; VDT, vibration detection threshold

**ESM Table 6. Correlation analysis of Pi:ATP and clinical/neurological variables.**

| | Right (r, p-value) | Left (r, p-value) | Mean (r, p-value) |
|---|---|---|---|
| Age, years | -0.176, 0.225 | -0.008, 0.958 | -0.119, 0.415 |
| Duration of Diabetes, years | 0.268, 0.104 | 0.295, 0.073 | **0.341, 0.036** |
| Body Mass Index, kg/m$^2$ | -0.079, 0.589 | **0.346, 0.015** | 0.149, 0.306 |
| Systolic blood pressure, mmHg | 0.066, 0.651 | 0.261, 0.070 | 0.194, 0.181 |
| HbA1c, mmol/mol | **0.356, 0.012** | 0.134, 0.359 | **0.309, 0.013** |
| Total Cholesterol, mmol/l | -0.127, 0.386 | -0.092, 0.530 | -0.135, 0.353 |
| ACR, mg/mmol | 0.054, 0.766 | -0.121, 0.502 | -0.024, 0.892 |
| Creatinine, µmol/l | 0.075, 0.610 | 0.220, 0.128 | 0.176, 0.226 |
| TCNS | 0.017, 0.910 | -0.090, 0.539 | 0.041, 0.778 |
| NIS-LL | **-0.003, 0.986** | -0.041, 0.781 | -0.025, 0.863 |
| Peroneal CMAP (mV) | -0.024, 0.876 | 0.010, 0.947 | -0.010, 0.947 |
| Peroneal MNCV (m/s) | -0.015, 0.925 | -0.215, 0.173 | -0.139 0.380 |
| Peroneal MNDL (msec) | 0.024, 0.877 | **0.323, 0.033** | 0.204, 0.184 |
| Tibial MNDL (msec) | 0.014, 0.924 | 0.091, 0.542 | 0.062, 0.677 |
| Sural SNAP (mV) | -0.167, 0.316 | -0.113, 0.498 | -0.176, 0.289 |
| NIS(LL)+7 | 0.032, 0.831 | -0.053, 0.722 | -0.010, 0.946 |
| ESC (µS) | 0.181, 0.224 | 0.140, 0.347 | 0.198, 0.183 |
| CDT, z-score | 0.057, 0.702 | 0.034, 0.821 | 0.055, 0.708 |
| WDT, z-score | -0.029, 0.844 | 0.003, 0.984 | -0.017, 0.908 |
| TSL, z-score | 0.085, 0.566 | -0.033, 0.826 | 0.037, 0.805 |
| CPT, z-score | 0.196, 0.181 | -0.070, 0.638 | 0.088, 0.554 |
| HPT, z-score | -0.069, 0.642 | 0.068, 0.648 | -0.006, 0.965 |
| PPT, z-score | -0.080, 0.590 | 0.027, 0.854 | -0.036, 0.807 |
| MPT, z-score | -0.256, 0.079 | 0.024, 0.869 | -0.179, 0.223 |
| MPS, z-score | -0.164, 0.266 | -0.096, 0.516 | -0.160, 0.277 |
| MDT, z-score | 0.014, 0.927 | 0.093, 0.528 | 0.061, 0.679 |
| VDT, z-score | 0.057, 0.702 | -0.016, 0.916 | 0.028, 0.851 |

CDT, cold detection threshold; CMAP, compound muscle action potential; CPT, cold pain threshold; DMA, dynamic mechanical allodynia; ESC, electrochemical skin conductance; HDT, heat detection threshold; HPT, heat pain threshold; MDT, mechanical detection threshold; MNCV, motor nerve conduction velocity; MNDL, motor nerve distal latency; MPS, mechanical pain sensitivity; MPT, mechanical pain threshold; NIS-LL, Neuropathy Impairment score of the lower limb; NIS(LL)+7, Neuropathy Impairment score of the lower limb plus 7 neurophysiological tests; PPT, pressure pain threshold; SNAP, sensory nerve action potential; TCNS, Toronto clinical neuropathy score; VDT, vibration detection threshold

**ESM Table 7. Correlation analysis between 1H- and 31P-MRS measures at right thalamus (r, p-value).**

| | PCr:ATP | Pi:ATP |
|---|---|---|
| NAA:Cho | -0.368, 0.023 | 0.218, 0.189 |
| Cho:Cr | 0.334, 0.040 | 0.0053, 0.754 |

Cho, Choline; Cr, Creatine; PCr, Phosphocreatine; Pi, Inorganic Phosphate

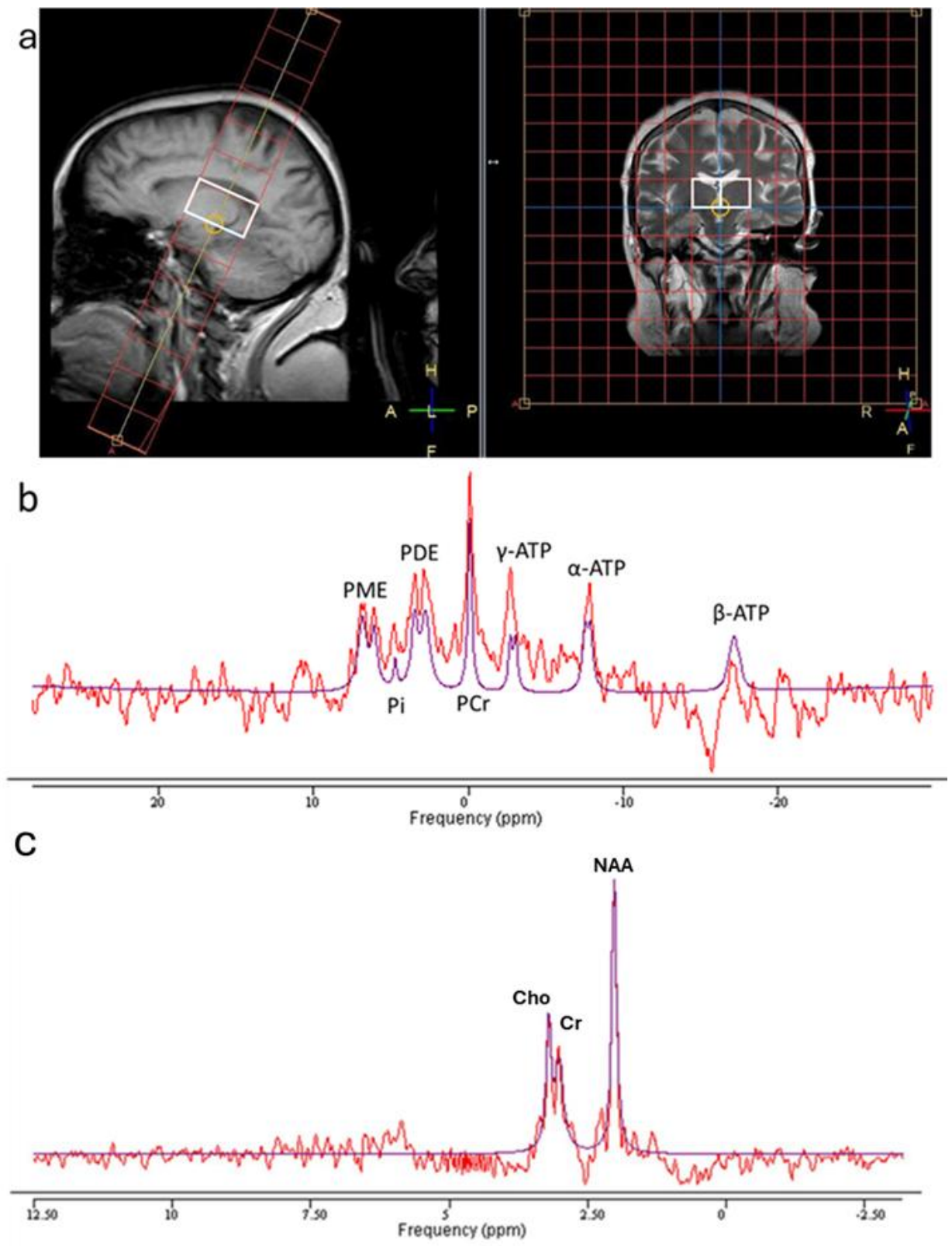


ESM Fig. 1.a) Voxel Positioning. b) Example spectra of 31P-MRS (red line) and fitting by AMARES (blue line). c) Example spectra of 1H-MRS (red line) and fitting by AMARES (blue line).

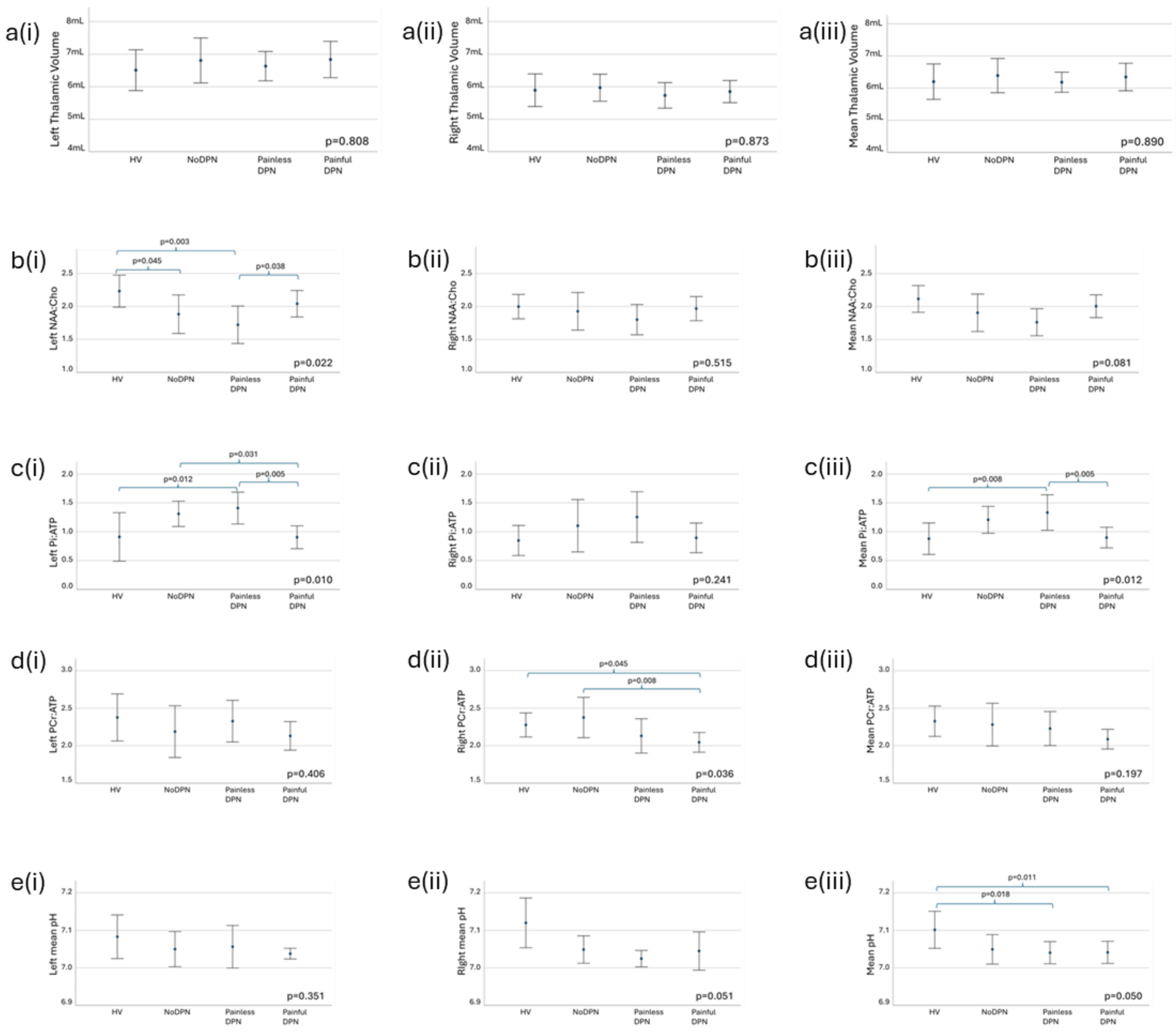


ESM Fig. 2. MR analysis for left (i), right (ii) and mean (iii) thalamic structural (a), $^{1}$H-MRS (b) and $^{31}$P-MRS (c-f) results. ATP, adenosine triphosphate; Cho, choline; Cr; creatine; NAA, N-acetyl aspartate; PCr, phosphocreatine; Pi, inorganic phosphate.
